# Expanded Availability of Intravenous Artesunate for the Treatment of Severe Malaria in the United States

**DOI:** 10.4269/ajtmh.19-0230

**Published:** 2019-03-29

**Authors:** Philip J. Rosenthal, Kathrine R. Tan

**Affiliations:** 1 Department of Medicine, University of California, San Francisco, California;; 2 Malaria Branch, Centers for Disease Control and Prevention, Atlanta, Georgia

Malaria remains one of the most important infectious diseases in the world. The WHO estimated 435,000 deaths from malaria in 2017, the large majority due to *Plasmodium falciparum*.^1^ In the United States, over the last 5 years with available data (2011–2015), between 1,517 and 1,925 cases of malaria have been reported annually, including about 300 cases of severe malaria and between five and 11 deaths each year. In non-immune individuals severe malaria most commonly presents as a rapidly progressive illness, with life-threatening dysfunction of the brain, lungs, kidneys, and other organs. Prompt administration of effective antimalarial therapy is an urgent, potentially life-saving priority.

Our oldest antimicrobial therapy is quinine, the active component of *Cinchona* bark, used to treat malaria since the 17th century, and available as a pure compound for this purpose for almost 200 years.^2^ For many years the standard of care to treat severe malaria was intravenous quinine. Subsequently, artemisinins were developed as potent, rapidly acting antimalarials, and two landmark studies showed intravenous artesunate to be superior to intravenous quinine, with improved survival in studies mostly of adults in Asia^3^ and of children in Africa.^4^ Intravenous artesunate is now recommended by the WHO, and is the international standard of care to treat severe malaria.^5,6^

In the United States, intravenous quinine was available from the CDC Drug Service until 1991, and then replaced by intravenous quinidine, the dextrorotatory stereoisomer of quinine, which was similarly effective for the treatment of severe malaria.^7^ Quinidine was routinely available in the United States as an antiarrhythmic, enabling its establishment as the standard of care to treat severe malaria. However, use of quinidine for severe malaria entailed a number of challenges. First, like quinine, quinidine has several concerning adverse effects. These include tinnitus, headache, nausea, dizziness, and visual disturbances (a constellation of symptoms termed cinchonism); less frequently hematologic abnormalities, hypoglycemia, and hypersensitivity reactions; and, with intravenous dosing, uncommon but life-threatening events including severe hypotension and dangerous arrhythmias. Due to these risks the drug requires intensive care for cardiac monitoring and is contraindicated for those with known hypersensitivity, severe cinchonism, hemolysis, or cardiac disease. Second, availability of intravenous quinidine in the United States decreased over time, as it was replaced by newer agents for cardiology indications. Physicians were at times challenged by difficulties in obtaining quinidine to treat dangerously ill malaria patients. Third, with the publication of the two landmark trials mentioned above, quinidine was proven demonstrably inferior to intravenous artesunate for the treatment of severe malaria.

To address the unavailability of artesunate, Walter Reed Army Institute of Research and the U.S. Army Medical Materiel Development Activity worked to develop intravenous artesunate for the United States. In 2007, the CDC obtained approval from the Food and Drug Administration (FDA) for an expanded access investigational new drug protocol (IND) for intravenous artesunate supplied by the U.S. Army Medical Research and Materiel Command.^8^ The IND is an FDA regulatory mechanism that gives an entity permission to procure and then release non-FDA approved drugs only under certain circumstances. It is unknown when artesunate will be approved by the FDA. The approval and subsequent commercial availability of intravenous artesunate is dependent on a drug company submitting an application to the FDA for review. Because intravenous quinidine was FDA approved and commercially available while artesunate was not, quinidine continued to be the first-line drug for severe malaria, while artesunate could be released under the IND only when quinidine was unavailable, contraindicated, poorly tolerated, or failing. To obtain artesunate, clinicians contacted CDC, and when eligibility was confirmed, the drug was distributed from the closest CDC Quarantine Station where it was stocked. This IND has provided intravenous artesunate for about 43 patients per year in the United States in recent years, and it has contributed to recovery from severe malaria in most of these patients.^9^ However, depending on an IND for availability of artesunate has significant limitations. Notably, the urgent treatment of severe malaria may be delayed since the drug is not readily available at the hospital. Delivery times for artesunate released under the IND were on average 7 hours (range: 3.5–15.5 hours).^10^ Not having artesunate as a readily available first-line treatment for severe malaria caused some distress and confusion among American providers, who questioned why the international standard of care to treat severe malaria was not routinely available in the United States.

In December 2017, the only manufacturer of intravenous quinidine in the United States announced that production of this product would be halted, with distribution to continue until the expiration date of the existing stock, the end of March 2019. With this news, the CDC has taken action to ensure availability of therapy for all those with severe malaria in the country. As of April 1, 2019, intravenous artesunate will be the first line drug for treatment of severe malaria in the United States. As artesunate is still not FDA-approved, the expanded access IND for intravenous artesunate will now allow CDC to release this drug for all cases of severe malaria. To meet the increased demand, CDC has imported additional artesunate and anticipates that there will be sufficient supply for all cases of severe malaria. To obtain the drug, clinicians treating patients with severe malaria should call the CDC Malaria Hotline (770-488-7788 Monday–Friday, 9 am–5 pm, Eastern time; outside these hours call 770-488-7100 and ask to speak with a CDC Malaria Branch expert). Artesunate will be prepositioned throughout the United States at select CDC quarantine stations and sent free of charge to the major airport closest to the requesting hospital. Delivery times will vary depending on the requesting hospital’s proximity to one of the storage sites. Since severe malaria can progress rapidly, it is appropriate to administer oral antimalarial therapy if delay in acquisition of artesunate is anticipated; acceptable therapies include initial treatment doses of artemether-lumefantrine (Coartem), atovaquone-proguanil (Malarone), quinine, or mefloquine. Artesunate is administered intravenously in four doses over 3 days, every 12 hours on the first day, and then daily. For adults and children ≥ 20 kg, the dose is 2.4 mg/kg, and for children < 20 kg, the dose is 3.0 mg/kg. After the intravenous artesunate course patients are given follow-on therapy with an oral agent when they can tolerate oral medications.

The availability of intravenous artesunate for all patients with severe malaria through an IND represents a step in the right direction. It is reassuring that the international standard is now also the clear standard of care in the United States. However, availability through an IND is not a long-term solution. Ideally, commercial availability will allow U.S. hospitals to stock this now first-line treatment. This will require a manufacturer of intravenous artesunate bringing the drug to the FDA for approval. Approval can be anticipated, as the drug has an excellent record of efficacy and safety around the world.^5^ Ultimately, achieving routine availability of intravenous artesunate will improve our ability to treat severe malaria in the United States.
